# Modifiable Lifestyle Risk Factors and Strategies for Slowing the Progression of Age-Related Macular Degeneration

**DOI:** 10.3390/vision9010016

**Published:** 2025-02-23

**Authors:** Khushi Saigal, Joshua E. Salama, Alfredo A. Pardo, Sebastian E. Lopez, Ninel Z. Gregori

**Affiliations:** 1 College of Medicine, University of Florida, 1600 SW Archer Road, Gainesville, FL 32610, USA; saigalkhushi@ufl.edu; 2 Division of Internal Medicine, Department of Medicine, University of Miami Miller School of Medicine, Miami, FL 33136, USA; joshsalama@med.miami.edu; 3 Department of Dietetics and Nutrition, Robert Stempel College of Public Health and Social Works, Florida International University, Miami, FL 33174, USA; apard056@fiu.edu; 4 Miami Veterans Administration Medical Center, Miami, FL 33125, USA; sebastian.e.lopez18@gmail.com; 5 Department of Ophthalmology, Bascom Palmer Eye Institute, University of Miami Miller School of Medicine, Miami, FL 33136, USA

**Keywords:** age-related macular degeneration, AMD, AREDS, AREDS2, diet, genetics, lifestyle factors, microbiome, nutrition, sleep, supplements, systemic health factors

## Abstract

Age-related macular degeneration (AMD) is a multifactorial disorder influenced by genetic, lifestyle, nutritional, and systemic health factors that contribute to increased oxidative stress and chronic inflammation in the retina. This article reviews the recent literature on modifiable lifestyle risk factors for the development and progression of AMD. Smoking (current and former), physical inactivity, prolonged sunlight exposure, as well as conditions such as diabetes, hypertension, cardiovascular disease, and obesity have all been associated with an increased risk of early AMD and its progression. The Age-Related Eye Disease Studies (AREDS and AREDS2) have shown that a specific combination of vitamins E and C, zinc, copper, lutein, and zeaxanthin can significantly reduce the risk of AMD progressing from dry to wet form. Additionally, adherence to a Mediterranean diet, rich in vegetables, fruits, legumes, whole grains, and nuts, has been linked to a lower risk of both early and late AMD. Emerging evidence suggests that these benefits may be influenced by the gut microbiota, as well as genetic and epigenetic factors. Further research into the interactions between these risk factors could pave the way for targeted therapies aimed at preventing or slowing AMD progression.

## 1. Introduction

Age-related macular degeneration (AMD) affects approximately one in eight individuals 60 years and above, making it the leading cause of irreversible blindness among the elderly in developed countries [1]. Current estimates suggest that 200 million people globally have AMD, and this number is expected to increase to nearly 300 million by 2040 [1]. By 2050, it is projected that 5.4 million Americans will be affected. Although most of those impacted are of Caucasian descent, intermediate dry AMD and exudative choroidal vasculopathy appear to be more prevalent in Asian and African American populations [2]. Because AMD is a chronic condition requiring ongoing long-term management, it poses a persistent public health challenge for both high- and low-income countries, leading to substantial socioeconomic consequences and increased healthcare costs [1].

The current review explores the intricate relationship between AMD and risk factors such as diet, smoking, physical activity, sleep, sunlight exposure, systemic health, the microbiome, and genetics. Given that AMD is one of the leading causes of vision loss among older adults worldwide, understanding its pathogenesis is critical for developing effective prevention and treatment strategies.

## 2. Nutrition and Diet

Although AMD pathogenesis encompasses a wide range of factors, extensive research has shown that nutrition and diet play a central role in the development and progression of AMD [3]. The primary mechanism linking diet to AMD is an alteration of oxidative stress, which is the imbalance between reactive oxygen species (ROS), such as hydrogen peroxide (H_2_O_2_) and superoxide (O_2_^−^), and antioxidants that stabilize or remove ROS [4]. The eye is one of the most oxygen-demanding organs; therefore, it is highly susceptible to oxidative stress [5]. Among the tissues of the eye, the retina has the highest oxygen demand, making it particularly susceptible to ROS formation, which has been associated with several major retinal disorders [6].

Consumption of foods containing high concentrations of simple sugars, such as glucose, leads to increased intracellular glucose concentration [7], which initially boosts the anabolic rate, enhancing the ability to synthesize intermediate metabolites by oxidative metabolism, resulting in enzymatic saturation due to substrate overload [7]. Approximately 95% of the oxygen entering mitochondria is used in oxidative processes, primarily in the Krebs cycle, while the remaining 5% reacts with water molecules to form hydrogen peroxide (H_2_O_2_) and other reactive species such as hydroxyl radicals (OH) and superoxide (O_2_^−^), collectively known as ROS [7]. Under normal conditions, ROS in the retina play a role in physiological signaling and protective mechanisms, mainly through the pro-survival extracellular signal-regulated kinase 1/2 pathway and endoplasmic reticulum stress signaling [8]. However, excessive ROS production leading to oxidative stress may contribute to the development of various retinal degenerative diseases, including diabetic retinopathy (DR), retinal vascular occlusion, retinitis pigmentosa (RP), age-related macular degeneration (AMD), glaucoma, and retinopathy of prematurity (ROP) [8].

A diet marked by excessive intake of saturated fats, simple sugars, high-fructose corn syrup, and sodium, combined with reduced consumption of antioxidants, dietary fiber, vitamins, and protein, has been correlated with increased levels of oxidative stress [7]. A prospective study demonstrated that a strong adherence to the Mediterranean diet, rich in leafy greens and red–orange–yellow fruits and vegetables, legumes, whole grains, nuts, and fish, lowers the risk of developing late-stage AMD [9]. Other prospective studies linked high-glycemic-index diets (those with carbohydrates that quickly and significantly raise blood glucose levels) to the development and progression of AMD [10]. Specific components of the diet, such as certain fruits and vegetables rich in antioxidants, including vitamins and carotenoids, are believed to help protect against disease progression [11].

To set some parameters, the Food and Drug Administration (FDA) and the Dietary Guidelines for Americans suggest that carbohydrate consumption should fall between 45% to 65% of an adult’s daily caloric intake [12]. For example, if a person’s daily caloric intake is 2000 calories, their carbohydrate consumption would range from 900 to 1300 calories. Focusing on whole grains, fruits, vegetables, and legumes is recommended to meet these carbohydrate needs healthily [13].

Ultimately, it has been shown that oxidative stress can lead to protein damage and chronic inflammation in the retina, damaging the retinal pigmented epithelium (RPE) and ultimately resulting in AMD [14]. It has also been shown that those with AMD have a lower mitochondrial oxygen consumption rate than healthy subjects, reflecting impaired energy production and increased oxidative stress in the retina, both of which contribute to retinal degeneration and disease progression [15]. Mitochondrial DNA (mtDNA) damage and deleterious mutations increase the risk of AMD, which is highly influenced by aging and external causes like oxidative stress. These findings are consistent with previous research by Wang and colleagues, who demonstrated that mtDNA damage accumulates with age in the RPE and choroid of mice and rats [8]. This accumulation is likely due to a decline in DNA repair capacity with aging, as evidenced by reduced expression of genes encoding key enzymes involved in repairing oxidative DNA damage, including 8-oxo-guanine-DNA glycosylase 1 (OGG1), MutY DNA glycosylase, and thymine DNA glycosylase [15].

## 3. Nutritive Versus Non-Nutritive Carbohydrates

Distinguishing between beneficial and harmful carbohydrate sources is essential for understanding their impact on health. In this context, healthy carbohydrates are defined as those derived from whole grains, whereas refined grains are classified as harmful to health [16]. Refined grains such as refined bread and rice, crackers, chips, and other ultra-processed foods undergo milling processes that remove the germ and bran layers, which are rich in nutrients and contain high levels of fiber, iron, B vitamins, and the antioxidant vitamin E [17]. Due to a lack of fiber, foods containing refined grains have been shown to significantly increase blood sugar levels because of the rapid digestion and absorption of simple sugars. In contrast, whole grains are not processed through milling and, thus, retain all layers (bran, germ, and endosperm) that contain higher fiber content. Fiber slows digestion, allowing for the gradual absorption of simple sugars and, thus, lowers glycemic index [18].

Notably, some patients may be concerned about the safety of consuming fruits due to their high fructose content. However, fruits are also rich in fiber and vitamins, which enhance their nutritional value and promote the gradual absorption of simple sugars [19]. Therefore, fruits and whole grains are generally considered safer and more nutritious options compared to refined grains and sugary foods [8].

## 4. Beneficial Effects of the Mediterranean Diet

There is strong evidence of positive health effects of the so-called Mediterranean diet, which consists of a high intake of vegetables, fruits, legumes, whole grains, and nuts; moderate intake of fish and alcohol; low consumption of red and processed meats and dairy products; and a preference for monounsaturated fats over saturated fats. The Mediterranean diet has been shown to have beneficial effects on cardiovascular health, lower rates of obesity, hypertension, diabetes, metabolic syndrome, and dyslipidemia, as well as better cognitive health and lower incidence of neurocognitive disorders such as Alzheimer’s disease [20]. There is also ample evidence of this diet’s positive effects on ocular health and the progression of AMD. A prospective cohort study of 4996 European participants 55 years and older revealed that after a mean follow-up of 9.9 years, those with high adherence to the Mediterranean diet showed a 41% reduced risk of developing advanced AMD compared to those with low adherence (hazard ratio (HR), 0.59; 95% confidence interval, 0.37–0.95; *p* = 0.04 for trend) [21]. Additionally, observational analyses of this trial indicated that greater adherence to this diet was associated with a reduced risk of mortality and chronic diseases [22]. Another prospective study, the Carotenoids Age-Related Eye Disease Study (CAREDS), involved 1325 American women aged 50 to 75 years who provided detailed dietary and lifestyle habit histories and underwent fundus photography as part of the Women’s Health Initiative. At an average 6-year follow-up, a higher score on a nine-point scale was associated with lower rates of early AMD, where a point was assigned for consuming greater than 75th percentile of each of the following: fruits, vegetables, whole grains, legumes, nuts, fish, and the ratio of monounsaturated to saturated fat; less than the 25th percentile for red meat servings; and light to moderate alcohol intake of 5 to 25 g per day [23].

Furthermore, a longitudinal study by Merle et al. based on food frequency questionnaires from 2525 subjects in the Age-Related Eye Disease Study (AREDS) reported a decrease in the progression to late AMD over a 13-year follow-up among those who followed the Mediterranean diet closely as calculated by the aMeDi score. A high aMeDi score (between 6 and 9) was significantly linked to a lower risk of progressing to advanced AMD after accounting for demographic, behavioral, ocular, and genetic factors (HR 0.74, 95% confidence interval (CI) 0.61, 0.91; *p*-trend = 0.007). Notably, the aMeDi score was associated with a decreased risk of developing advanced AMD in individuals who carried the non-risk (T) allele of the CFH Y402H gene (*p*-trend = 0.0004, *p*-interaction = 0.04) [9]. In another study, Merle et al. examined two European studies, the Rotterdam Study I (RS-I) and the Alienor Study, finding a 41% reduction in the risk of progressing to late AMD with higher adherence to the Mediterranean diet (HR 0.59; 95% CI 0.37–0.95; *p* < 0.04 for trend) [21]. A study by Chiu et al. analyzed dietary patterns in 4088 participants of the AREDS study and classified them according to their food composition data collected with a 90-item food frequency questionnaire [24]. They ranked the participants according to how closely their diets resembled major dietary patterns—the Western pattern (high intake of red meat, processed meat, high-fat dairy products, French fries, refined grains, and eggs) and the Oriental pattern (higher intake of vegetables, legumes, fruit, whole grains, tomatoes, and seafood). In the Oriental pattern group (which closely resembles the Mediterranean diet), the odds ratio (OR) was 0.74 (95% CI 0.59–0.91; *p* = 0.01) for early AMD and 0.38 (0.27–0.54; *p* < 0.0001) for advanced AMD. On the other hand, the Western pattern group showed a significantly higher OR of 1.56 (1.18–2.06; *p* = 0.01) for early AMD and 3.70 (2.31–5.92; *p* < 0.0001) for advanced AMD [24].

Retrospective analysis of data from two controlled clinical trials, Age-Related Eye Disease Study (AREDS) and Age-Related Eye Disease Study 2 (AREDS2) focusing on participants who did not have late AMD at the start (totaling 7756 participants and 13,204 study eyes) reported that over a median follow-up of 10 years, 34.0% progressed to late AMD, defined as either fovea-involving geographic atrophy (GA) or neovascular AMD [25]. Closer adherence to the Mediterranean diet was associated with a lower risk of progression to large drusen and late AMD, with a greater reduction in the risk of developing GA than neovascular AMD after adjusting for age, sex, smoking status, total caloric intake, and body mass index. The differences in HRs were more pronounced for GA compared to neovascular AMD. The HRs for progression in tertile 3 (highest) versus 1 (lower adherence) were 0.78 (*p* < 0.0001) for late AMD, 0.71 (*p* < 0.0001) for GA, and 0.84 (*p* = 0.005) for neovascular AMD [25]. Higher fish intake was associated with decreased risk of GA and neovascular AMD. Notably, genotype analysis examining the CFH protective allele and ARMS2/HTRA1 risk alleles showed that the association between higher Mediterranean diet adherence and decreased late AMD was found only in those with CFH protective alleles. This suggests that the benefits of the Mediterranean diet may vary based on genotype, and additional preventive strategies may need to be tailored according to an individual’s genetics [25].

## 5. Age-Related Eye Disease Study (AREDS) and Age-Related Eye Disease Study 2 (AREDS2) Nutritional Supplements

The two pivotal long-term, prospective randomized controlled trials that established the benefit of dietary supplements in lowering the risk of developing advanced forms of AMD are The Age-Related Eye Disease Study (AREDS) and The Age-Related Eye Disease Study 2 (AREDS2) [26]. In 2024, the most comprehensive evidence to support recommendations regarding nutritional supplementation for patients at risk of developing advanced forms of AMD comes from these two prospective trials.

**Figure 1:**
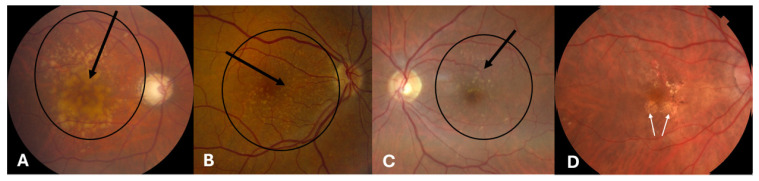
Examples of category 3 age-related macular degeneration. (**A**) Shows multiple large drusen. (**B**,**C**) Show several large drusen and many intermediate drusen. (**D**) Demonstrates non-fovea involving geographic atrophy without frank drusen in the macula. Drusen are indicated by the black arrows depicted in (**A**,**B**,**D**), and non-fovea involving geographic atrophy is indicated with white arrows in (**D**).

The AREDS trial enrolled 3609 participants with various stages of AMD and provided level 1 evidence that a combination of 500 mg vitamin C, 400 international units (IU) vitamin E, 15 mg beta-carotene, 80 mg zinc, and 2 mg copper significantly reduced the 5-year risk of moderate visual loss (≥15 ETDRS letters) and development of advanced AMD by 25% in patients with at least intermediate AMD, defined by this study as those with Category 3 or Category 4 AMD [27]. Advanced AMD was defined as the presence of either fovea-involving geographic atrophy (GA) or choroidal neovascularization. Category 3 was defined as the absence of advanced AMD in both eyes and at least one eye with visual acuity of 20/32 or better with at least one large druse (≥125 μm, i.e., greater than or equal to the diameter of an average normal retinal vein at the disc margin), extensive intermediate drusen (approximately 20 intermediate (63–124 μm) drusen if soft drusen are present or at least 65 intermediate drusen if soft drusen are absent), non-fovea involving GA, or any combination of these features (Figure 1). Category 4 was defined as one eye (the study eye) with visual acuity of 20/32 or better and no advanced AMD features and the fellow eye with either advanced AMD (fovea-involving GA or neovascular membrane) or visual acuity of less than 20/32 due to AMD. Only participants with Category 3 or 4 features randomized to antioxidants plus zinc had a significant reduction in the risk of moderate visual loss in 5 years. Notably, beta-carotene was shown to increase the risk of lung cancer in cigarette smokers. Based on these findings, the AREDS study recommended supplementation with the AREDS formulation of antioxidants plus zinc to patients with intermediate AMD consisting of numerous intermediate-size drusen, at least one large druse, or noncentral GA in one or both eyes or advanced AMD (neovascular or fovea involving GA) or vision loss due to AMD in one eye. Due to a lack of demonstrated evidence, the AREDS formulation is not recommended for patients with early AMD or those without AMD.

Later, the Age-Related Eye Disease Study 2 (AREDS2), an extension of the AREDS trial, enrolled 4203 participants to examine the effects of beta-carotene deletion (given the risk of lung cancer in smokers), zinc content reduction in the original AREDS formulation from 80 mg to 25 mg (due to the risk of anemia, decreased high-density lipoprotein cholesterol, and dyspepsia), and the possible additional benefit of adding omega-3 fatty acids (eicosapentaenoic (EPA) and docosahexaenoic (DHA) acid) and/or macular xanthophylls (lutein and zeaxanthin) [28].

The AREDS2 data indicated that the progression rate to advanced AMD was similar across the study groups and the placebo group after a median follow-up of 5 years: 31% for the placebo group, 29% for the lutein-zeaxanthin group, 31% for those taking DHA plus EPA, and 30% for the combination of lutein, zeaxanthin, DHA, and EPA [29]. Further subgroup analyses revealed a protective effect of lutein and zeaxanthin in the lowest quintile (those with the lowest dietary intake) but not in the higher quintiles, no beneficial or harmful effects of DHA with EPA, and no impact of beta-carotene elimination or the use of a lower-dose zinc formulation on progression to advanced AMD [29]. None of the studied nutrient combinations affected the development of moderate visual loss (≥15 ETDRS letters). Furthermore, directly comparing lutein/zeaxanthin to beta-carotene demonstrated that lutein/zeaxanthin provided a significantly stronger protective effect against progression to late AMD (HR 0.82, *p* = 0.02), particularly for the development of neovascular AMD (HR 0.78, *p* = 0.01) but not for the development of central GA (HR 0.94, *p* = 0.67) [30].

Historically, the AREDS trial also showed no significant benefit of antioxidants or zinc on the development or progression of any or central GA with a median time to progression from non-foveal GA to sub-foveal GA of 2.5 years [31]. However, a recent post hoc analysis of AREDS and AREDS2 data in over 1200 participants with GA looked at whether oral micronutrient supplementations slowed area-based and proximity-to-the fovea-based progression of GA measured on color fundus photographs [32]. In AREDS, GA progression toward the central macula was significantly slower in participants randomized to antioxidants versus no antioxidants (50.7 µm/year vs. 72.9 µm/year, *p* = 0.012), but not comparing zinc versus no zinc. In AREDS2, GA progression toward the central macula was significantly slower in participants assigned to AREDS antioxidants with or without β-carotene and randomized to lutein/zeaxanthin versus none (for the ARED2 with no β-carotene group, 80.1 µm/year with lutein/zeaxanthin vs. 114.4 µm/year on none, *p* = 0.011). Supplemental analysis revealed that GA progression toward the central macula was slower in participants randomized to lutein/zeaxanthin alone versus placebo (*p* = 0.039) and in those randomized to lutein/zeaxanthin and DHA/EPA versus placebo (*p* = 0.040). In AREDS, area-based progression was not significantly different with randomization to antioxidants versus none. In AREDS2, area-based progression was significantly slower in participants randomized to β-carotene than no β-carotene (0.264 mm/year vs. 0.301 mm/year, *p* = 0.009). There was no difference in other comparisons. When comparing visual acuities, the rate of visual acuity decline in AREDS was slower in participants randomized to antioxidants than no antioxidants (*p* = 0.007). In AREDS2, a borderline slower visual acuity decline was seen in participants randomized to lutein/zeaxanthin and in those randomized to β-carotene and also to low zinc. However, because β-carotene competes with lutein/zeaxanthin for intestinal absorption, does not slow progression toward the center on its own, and increases the risk of lung cancer in those with a history of smoking (see below), a formulation with vitamin C and E, and lutein/zeaxanthin without β-carotene would be most recommended [32].

In summary, these new analyses of AREDS showed that oral antioxidants (vitamin C, vitamin E, and β-carotene) led to approximately 36% slower GA progression toward the central macula. Similarly, in AREDS2, oral lutein/zeaxanthin supplementation led to approximately 35% slower GA progression toward the central macula. The effect of lutein/zeaxanthin was additive to that of vitamins C and E. Interestingly, while the AREDS population with noncentral GA showed treatment effect with antioxidants for progression toward the center but not area-based GA progression, the incident noncentral GA (new onset) showed the opposite effect. The natural distribution of antioxidants and lutein/zeaxanthin in the central and paracentral macula is the likely explanation of why vitamin C, vitamin E, and carotenoids supplements had a positive effect on the progression toward the central macula rather than peripheral macula or area-based calculations and may be important for foveal sparing from GA in AMD.

However, a very recent post hoc analysis of data from OAKS and DERBY clinical trials of pegcetacoplan for GA (presented by Nathan C. Steinle at Angiogenesis 2025 meetings in February 2025) showed no effect of AREDS/AREDS2 on the growth of GA toward the fovea and no effect on overall GA growth in untreated or treated participants, i.e., no effect on pegcetacoplan efficacy [33]. The data analyzed were based on measurements of GA in fundus autofluorescence images by two independent graders, which is a much more sensitive method for the evaluation of GA compared to fundus photographs. Thus, these conflicting results highlight the need for a prospective trial to study the potential effect of AREDS/AREDS2 supplementation on GA growth.

Notably, there were more lung cancer cases in the beta-carotene group compared to the group without beta-carotene (2.0% vs. 0.9%, *p* = 0.04), with 91% of lung cancer cases among former smokers [29]. Serious adverse events and mortality rates were similar among the various groups. The AREDS2 study concluded that, although adding lutein plus zeaxanthin, DHA plus EPA, or both to the original AREDS formulation did not show additional benefits, lutein and zeaxanthin could replace β-carotene in the original AREDS formulation. This substitution would help mitigate the increased risk of lung cancer in current and former smokers [29].

The 10-year follow-up of the AREDS2 cohort consisting of 3882 participants, who received AREDS2 supplements with lutein/zeaxanthin, vitamins C and E, and zinc plus copper for 5 years after the conclusion of AREDS2 trial, assessed the 10-year risk of developing lung cancer and late AMD [26]. At 10 years, the odds ratio (OR) of having lung cancer was 1.82 (*p*  = 0.02) for those randomly assigned to β-carotene and 1.15 (*p*  =  0.46) for lutein/zeaxanthin, with β-carotene nearly doubling the risk of lung cancer. The HR for progression to late AMD for low vs. high zinc was 1.04 (*p*  =  0.49), and the HR for no β-carotene vs. β-carotene was 1.04 (*p*  =  0.48). Comparing lutein/zeaxanthin with no lutein/zeaxanthin, the HR was 0.91 (*p* = 0.02), ω-3 fatty acids with no ω-3 fatty acids HR was 1.01 (*p*  =  0.91). The direct analysis of lutein/zeaxanthin vs. β-carotene showed an HR of 0.85 (*p*  =  0.02), indicating a beneficial association of lutein/zeaxanthin with late AMD progression. Thus, the AREDS2 formulation, which consists of 10 mg lutein, 2 mg zeaxanthin, 80 mg zinc oxide, 2 mg cupric oxide, 500 mg vitamin C, and 400 IU vitamin E, is currently recommended for patients with fundus findings corresponding to AMD category 3 and category 4 features (Table 1) [26].

Table 1 lists the dosages for supplements recommended by the Age-Related Eye Disease Study 2.

## 6. Curcuma-Based Nutritional Supplements (Turmeric)

Recently, consumption of curcuma-based nutritional supplements was found to be associated with a lower risk of developing age-related macular degeneration. Curcumin, an active ingredient of turmeric, is isolated from the plant Curcuma longa and is used as a spice or a medicinal remedy throughout South Asia [34]. The pharmacological activities of curcumin include antioxidant and anti-inflammatory effects, which have been linked to the reduction in free radical production and inflammatory marker downregulation [35]. A recent retrospective cohort study using a large database of electronic health records from patients aged 50 and older without AMD showed a 77% risk reduction in developing nonexudative AMD (relative risk [RR] 0.23; *p*  <  0.001), an 89% reduction in developing advanced nonexudative AMD with GA (RR 0.11; *p*  <  0.001), a 72% reduction in developing exudative AMD (RR 0.28; *p*  <  0.001), a 54% reduction in developing blindness (RR 0.46; *p*  <  0.001), and a reduced need for intravitreal anti-VEGF therapy (RR 0.15; *p*  <  0.001) in 66,804 patients taking a curcuma-based nutritional supplement (CBNS) compared to 1,809,440 patients not taking CBNS. When looking at patients with early nonexudative AMD, CBNS prescription was associated with lower rates of developing advanced nonexudative AMD (RR 0.58, *p* < 0.001). All races and ethnicities were included—Asian, Black or African American, Hispanic or Latino, White, and unknown [36].

Additionally, in a small retrospective study comparing 18 patients with exudative AMD receiving intravitreal anti-VEGF injections and daily oral curcumin nutritional supplement (CNS) with 24 age-matched controls undergoing only intravitreal injections (both groups received initial 3 monthly loading doses), the median best-corrected VA improved significantly (*p* < 0.05), and the total number of injections was significantly lower in those receiving oral CNS (median of 4 versus seven injections, *p* < 0.05) [37]. Based on these intriguing retrospective data, future prospective trials are warranted to explore curcumin nutritional supplements as a potential adjuvant to the prevention and treatment of AMD.

## 7. Tobacco Smoking

Lifestyle is the most influential modifiable factor for overall human health. A healthy lifestyle can significantly decrease the risk for various diseases, including AMD. Although lifestyle is a broad term that encompasses exercise, diet, occupation, and smoking status, an overall unhealthy combination of these factors has been strongly linked to the development of AMD [38]. The primary lifestyle choices that increase the likelihood of developing AMD are tobacco smoking and overall physical health and activity.

Smoking releases toxic substances, including nicotine, heavy metals, and tar. These substances generate free radicals, more specifically ROS, which concentrate in the retina and lead to oxidative stress [39]. The macula is particularly sensitive to oxidative stress compared to other structures of the eye [40]. Cigarette smoking has been shown to promote both molecular and pathological changes that create an ocular environment more likely to develop AMD [39]. These factors include endothelial dysregulation, vascular inflammation, toxic and oxidative damage to retinal cells, and histopathological changes such as RPE cell apoptosis and basal deposits, as well as debris accumulations in Bruch’s membrane. Suñer et al. found a significant correlation between the concentration of nicotine in water given to mice and increased vascularity and size in the choroidal neovascularization (CNV) of exudative AMD [39]. This study concluded that the size of vascular margins (1.543 ± 0.49 DA vs. 0.884 ± 0.23 DA, *p* < 0.0124), cellular margins (2.096 ± 0.44 DA vs. 1.292 ± 0.29 DA, *p* < 0.0028), as well as maximal thickness (9.77 ± 1.53 pixels vs. 7.52 ± 1.54 pixels, *p* < 0.0187) of CNV were significantly larger in the eyes of mice who were treated with nicotine than those who were not [39].

In a prospective cohort study, the Nurses’ Health Study, of over 31 thousand registered nurses aged 50–59 years, current smokers of 25 or more cigarettes per day had a RR of AMD of 2.4 (95% CI 1.4–4.0), and past smokers had a two-fold increased risk (RR 2.0; 95% CI 1.2–3.4) compared with never-smokers [41]. Little reduction in RR was documented even 15 or more years after quitting. The risk of AMD also significantly increased with an increasing number of pack-years smoked. These results were similar to nonexudative and exudative types of AMD in women [41].

A prospective cohort study, the Physicians’ Health Study, of over 21 thousand US male physicians without a diagnosis of AMD at baseline, showed that current smokers of 20 or more cigarettes per day had an increased risk of AMD relative to never-smokers (RR 2.46; 95% CI 1.60–3.79), while past smokers had a RR of 1.30 (95% CI 0.99–1.70) [42]. Current smokers of fewer than 20 cigarettes per day demonstrated a nonsignificant 26% increased risk of AMD [42]. Thus, the risk for AMD is directly proportional to years smoked and pack-years, demonstrating a dose-response relationship between cigarette smoking and AMD pathogenesis [41].

A population-based longitudinal cohort study, the Beaver Dam Eye Study, examined 4926 participants aged 43 to 84 years who were followed over a 15-year period [43]. This study graded stereoscopic color fundus photographs and determined that current smokers, compared to those who never smoked, had an increased risk of early AMD (OR 1.47, *p* = 0.01) and of AMD progression (OR 1.43, *p* = 0.02) [43]. After controlling for age, sex, and baseline AMD severity, current smoking was associated with an approximately 45% increased odds of developing early AMD or AMD progression during the 15-year period [43]. No relationship was detected between smoking status and incidence of either exudative AMD or GA, but it was not conclusive due to the small number of these findings in this study [43]. However, a population-based cohort study from three countries (Australia, Netherlands, and the United States) reported that current smoking was associated with an increased incidence of GA and late AMD (ORs relative to nonsmokers: 2.83 and 2.35, respectively; ORs relative to past smokers: 2.80 and 1.82, respectively [44].

Multivariant analysis of the Beaver Dam Offspring Study involving 2810 participants aged 21 to 84 years reported that higher pack-years smoked was associated with an increased OR of developing early AMD, specifically, the OR was 1.31 comparing 0 packs versus 1–10 packs, and the OR was 1.67 comparing 0 packs versus ≥11 packs [45]. Notably, smoking has been linked to all the retinal alterations associated with early AMD, further proving cigarette smoking’s association with the pathogenesis of AMD [45].

## 8. Physical Activity

Physical activity levels are another prevalent lifestyle factor that has been associated with the development and progression of AMD. Regular exercise increases antioxidant enzyme activity and increases resistance to oxidative stress, which contributes to aging and AMD [46]. In a meta-analysis of nine studies of white subjects aged 30–97 years conducted in US, Europe, and Australia, after controlling for age, sex, and smoking, it was concluded that regular moderate physical activity was related to rates of both early AMD (eight studies, *n* = 38,112, OR 0.92, 95% CI 0.86–0.98) and late AMD (seven studies, *n* = 28,854, OR 0.59, 95% CI 0.49–0.72) [47]. In a preclinical study, age-matched mice housed in cages equipped with running wheels allowing for a preconditioning period exhibited a 41% reduction in laser-induced choroidal neovascularization compared to sedentary mice. Neovascular lesions of exercise-trained mice also exhibited significantly lower macrophage staining and Vegfa and Ccl2 mRNA expression, offering a potential connection behind the effects of exercise on AMD-relevant phenotypes in experimental models [48].

## 9. Sunlight Exposure

Excessive sunlight exposure is also suspected to play a role in the development of AMD, although few studies so far have shown an association between AMD and exposure to sunlight [49]. Several mechanisms by which light causes damage in the retina have been proposed [49].

Short-wavelength light bleaches rhodopsin and causes subsequent rod destruction, damages cones, and impairs mitochondrial metabolism [49]. UV radiation leads to oxidative stress and reactive oxygen species (ROS) production, which induce apoptosis, DNA damage, mitochondrial dysfunction, and alteration of RPE cells, all of which are implicated in the pathogenesis of AMD. The RPE cells selectively absorb the lower wavelengths of light, which protects the retinal cells; however, with increasing age, the RPE accumulates granules that contain lipofuscin and melanin [50]. Lipofuscin plays a central role in the pathogenesis of AMD, as its accumulation leads to the peroxidation of lipids, enzyme inactivation, and ROS generation [50]. These changes lead to lysosomal damage of RPE, complement activation and local inflammation, and promote drusen formation. The Beaver Dam Eye study found that there was a significant association between the time spent outside and the development of early AMD (OR 2.26; 95% CI 1.06–4.81) [51]. Similar to the role of visible and UV rays on AMD, further studies are needed to find conclusive evidence that blue light may have an effect on AMD development. Although blue light has been implicated in the pathogenesis of AMD, epidemiological evidence is equivocal.

## 10. Systemic Health Factors

AMD is a multifactorial disease, and systemic health factors have been associated with the development and progression of AMD. The main modifiable health conditions linked to AMD are diabetes, heart disease, hypertension, and obesity; however, research in this arena is limited [52]. The presence of AMD has been found to be a risk factor for cardiovascular disease and particularly a risk of stroke in patients with late AMD [53]. A meta-analysis demonstrated that late AMD is a more potent predictor for cardiovascular disease (including coronary heart disease and stroke) than early AMD and that stroke is more tightly associated with AMD than coronary heart disease [53]. On the other hand, the Beaver Dam Study did not demonstrate any association between a history of stroke or heart attack and the incidence or progression of AMD [54].

Both exudative and nonexudative AMD have also been associated with the risk of heart failure independent of hypertension, diabetes mellitus, hyperlipidemia, coronary artery disease, and chronic kidney failure [55]. These diseases are thought to be linked to AMD because they all damage the integrity of the retinal and choroidal vasculature and cause structural changes in the RPE, photoreceptors, and Bruch’s membrane [56]. In a cohort study of 1,768,018 diabetics over 50 years old who were followed for approximately 6 years in South Korea National Health Insurance Service, 7331 participants developed exudative AMD [56]. Those who had diabetes for 5 or more years had a greater risk of developing AMD with an HR of 1.13 (95% CI 1.07–1.18). Furthermore, insulin use (HR 1.16) and the presence of vision-threatening diabetic retinopathy (HR = 1.40) were associated with an increased risk of exudative AMD, particularly in those younger than 65 years of age [56]. Other studies, including the Barbados Eye Study [57], Women’s Health Initiative Study [54], AREDS [58], the Beaver Dam Eye Study [59], and the European Eye Study [60], have corroborated these findings, showing a significant association of diabetes with late and advanced AMD.

Diabetes and obesity can also cause inflammation and increased oxidative stress in the retina. Oxidative stress and damage to vasculature can compromise the delivery of protective carotenoid pigments to the macula [61]. Carotenoids, lutein, and zeaxanthin are concentrated in the central macula and function as antioxidants and blue light filters. By controlling other factors such as age, gender, smoking, heavy drinking, and vitamin use, the Beaver Dam Eye Study determined that higher systolic blood pressure was associated with the 10-year incidence of RPE depigmentation (a sign of early AMD) and exudative macular degeneration while higher pulse pressure was also linked to the progression of AMD [62]. Higher high-density lipoprotein (HDL) cholesterol was associated with GA [62]. Still, the research regarding systemic health factors and their correlation to AMD is limited; thus, further research is needed to confirm that these systemic health factors significantly affect the risk of acquiring AMD.

## 11. BMI and Obesity

An analysis of 7 prospective cohort studies identified 1613 cases of AMD among 31,151 pooled subjects and determined that the risk of developing AMD for those who were obese increased by 32% [63]. Notably, for every increase of 1 kg/m^2^ in BMI within overweight and obese BMI ranges, the risk for AMD increased by 2% [63]. A reduction in waist-to-hip ratio (WHR) of 3% or more was linked to a 29% lower likelihood of developing any AMD (OR 0.71; 95% CI 0.52–0.97). This association was especially significant among participants who were obese at the start, where a decrease in WHR corresponded to a 59% lower chance of AMD (OR 0.41; 95% CI 0.20–0.82) [64]. A large epidemiological study, the Melbourne Collaborative Cohort Study, conducted with 21,287 participants aged 40–69 years, demonstrated abdominal obesity as an independent risk factor in men for early AMD (OR 1.13; 95% CI 1.01–1.26; *p* = 0.03) and late AMD (OR 1.75; 95% CI 1.11–2.76; *p* = 0.02) [65]. Interestingly, in women, there was an inverse association for early AMD with all adipose measures studied (OR 0.89–0.93, *p* = 0.002–0.02) and no association for late AMD [65].

## 12. Microbiome

The microbiome is a community of microorganisms along with all their genetic material [66]. The human microbiota is the collection of these microorganisms, including bacteria, viruses, archaea, and Eukarya, that colonize the human body and play a role in health and disease, with the gut microbiota being particularly significant [66]. The gut microbiome is an integral part of maintaining homeostasis, playing roles in digestion, nutrient harvesting and bioavailability, vitamin biosynthesis, and immune function. Various environmental factors, such as diets high in saturated fats and sugars, are shown to cause gut dysbiosis, a proinflammatory state in which typical gastrointestinal tract flora is disrupted [67]. An imbalance in the gut microbiome has multiple deleterious effects on normal human physiology, including activation of inflammatory mechanisms, which is implicated in the development of diseases such as colitis, type 1 diabetes mellitus, rheumatoid arthritis, multiple sclerosis, respiratory diseases, mental and psychological diseases, and ocular diseases, including uveitis, dry eye, and AMD [68].

A study comparing the microbiomes of patients with advanced AMD versus controls demonstrated the predominance of bacteria associated with inflammatory disease states, such as Prevotella, Holdemanella, and Desulfovibrio, in AMD patients [69]. Neovascular AMD patients showed an increase in Anaerotruncus, Oscillibacter, Eubacterium ventriosum, and Ruminococcus torques and a decrease in Bacteroides eggerthii, with AMD patients’ intestinal microbiomes being enriched in genes involved in the metabolism of amino acids and decrease in genes involved in elongation of fatty acids and carotenoid biosynthesis [70].

Preclinical studies comparing germ-free mice (without any microbes) with conventional microbiome mice revealed a link between microbiome and retina, showing a differential expression of multiple genes in these mice populations, including CHF (complement factor H), HIF-1 (hypoxia-inducible factor), VEGF (vascular endothelial growth factor), AMPK (5′ ANO-activated protein kinase), among others, which are implicated in AMD pathogenesis [71,72]. Furthermore, it has been shown that diet alters microbiome composition and host gene expression. Mice fed a high-calorie, high-fat, simple-sugar diet (Western diet) show a decrease in the diversity and alterations of their gut microbiota, leading to an increased capacity to break down indigestible dietary polysaccharides and overall alterations of metabolism of the animals, leading to increased gut permeability and systemic inflammation [73]. Mice on a high-fat diet exhibited upregulation in the expression of genes involved in retinal inflammation, angiogenesis, and altered RPE function compared to mice fed a normal diet, demonstrating a link between diet, micronutrients, microbiome, genomics, and risk of ocular diseases, a concept knows as a “gut–retina axis” [74]. Inflammatory genes such as tumor necrosis factor receptor superfamily member 13B (Tnfrsf13b) and prostaglandin-endoperoxide synthase 2 (Ptgs2), as well as mediators of angiogenesis, such as Vegfc and angiopoietin genes (Angpt1, Angpt2, Angptl2), were upregulated in mice fed a high-fat diet [75].

Another study reported photoreceptor cell and RPE cell damage and subretinal deposits similar to the changes in dry AMD associated with gut microbial and metabolomic changes in mice fed a high-glucose diet [76]. Although the mechanisms connecting gut microbiota to retinal health are still being elucidated, future research on specific microorganisms and their relationship to AMD may help clinicians design new treatments and dietary interventions that may prevent or modulate the course of AMD and other ocular conditions.

## 13. Genetics and Epigenetics

In addition to the various environmental factors that play a role in AMD and its progression, human genes and their epigenetic modifications have significant correlations for susceptibility [77]. It has been shown that individuals with a sibling with AMD have a 12-fold increased risk of AMD, which is even higher if a parent is affected [78]. Many genetic mutations confer an increased risk independent of the environment, while others are modified only when exposed to extrinsic risk factors, such as tobacco smoke [77]. Multiple genetic factors influence the development and progression of AMD. These include genes within the complement system (CFH, C3, CFB/C2, CFI, C9, CD46), extracellular matrix genes (TIMP3, MMP2, MMP9), angiogenesis associated genes (VEGFA, TGFBR1), lipid metabolism genes (APOE, LIPC, CETP, ABCA1), apoptosis-associated genes (IER3, TNFRSF10A, CTRB2), ARMS/HTRA1 locus, PLEKHA, ARMS/LOC387715, and HTRA1), and inflammatory genes involved in the immune response (IL1B1, TNFRS1A, TNFRS1B) [79,80]. Some variants increase AMD risk, while others reduce it. These mutations, many of them single nucleotide polymorphisms (SNPs), have been found in over 40 different loci in the human genome [77]. Some of the most commonly mutated genes include NOS2A and CFH, the former of which confers a significantly increased risk of AMD when the patient has a history of smoking [81]. SNPs in the CFH gene, such as rs1061170 CC and rs2274700 CC, have a strong correlation with AMD development [77]. While a CFH mutation is associated with an increased risk for bilateral geographic atrophy, genes HTRA1/LOC387715 are more likely to be seen in patients with choroidal neovascularization in wet AMD [38]. Recently, the TAB2 rs237025 allele A was found to be a significant risk factor for both early and exudative AMD, with gender-specific associations noted in females with exudative AMD, indicating distinct pathogenic mechanisms. Although IKBKB rs13278372 and serum IKBKB protein levels were not significantly associated with AMD development, the presence of the A allele of IKBKB rs13278372 correlated with a poorer response to anti-VEGF therapy, suggesting its potential as a marker for treatment outcomes. Furthermore, the IKBKG rs2472395 variant was protective against early AMD in males and exudative AMD in females [82].

In recent years, research has shown an interplay between diet and genetic modification, or epigenetics, in AMD development and progression. The Mediterranean diet contains a wide variety of healthy micro- and macro-nutrients, many of which are included in the AREDS supplementation guidelines. Being high in fish protein, legumes, whole grains, and vegetables, this diet can significantly reduce AMD progression in subjects with specific genotypes [38]. Analysis of data from the AREDS study showed that subjects with the CFH rs10922109 mutation who followed a Mediterranean diet were less likely to develop late AMD (*p* = 0.01), GA (*p* = 0.003) and neovascular AMD (*p* = 0.026) than those who followed a standard diet [25]. Notably, in the AREDS, adherence to the Mediterranean diet and fish consumption were associated with a lower risk of late AMD and GA only in participants with at least one protective CFH allele [25]. These data illustrate that genotypes may play a role in the modification of environmental and lifestyle effects on AMD risk in patients.

Genetic testing for AMD is a topic of considerable debate; however, there is insufficient evidence to recommend genetic testing in clinical practice as of 2024 [38]. At this time, there are no prospective studies evaluating whether genetic testing can predict the progression of AMD or affect the efficacy of dietary interventions and other treatments, such as intravitreal anti-VEGF injections or the oral use of AREDS2 supplements. Retrospective studies analyzing these aspects have produced mixed outcomes. Furthermore, prediction models in various papers have not demonstrated significant benefits from including genetic information in predicting AMD progression. The baseline severity of AMD is a strong indicator of disease progression and is further influenced by environmental factors such as smoking and lifestyle habits. The American Academy of Ophthalmology does not currently recommend genetic testing for AMD [38].

Diet is one of the many environmental factors that have the ability to elicit epigenetic alterations. Age, sunlight exposure, smoking, and obesity have also been found to have epigenetic components, emphasizing the need for further research in this area [83].

## 14. Sleep Duration

Sleep deficiency has been identified as a leading factor in many health issues, including cognitive function, cardiovascular health, metabolic regulation, and immune function, and there are some emerging yet inconclusive data in AMD. One case-control study consisting of 165 subjects (57 patients with neovascular AMD and 108 controls) who completed a questionnaire on sleep quality and quantity reported an increased risk of AMD associated with short sleep duration: less than 6 h, the OR was 3.29 (95% CI 1.32–8.27); 2.25 for 6–7 h (95% CI 0.80–6.32); 1.39 for more than 8 h (95% CI 0.53–3.73), compared with the reference category of 7–8 h [84]. After adjustment for confounders, a sleep duration of less than 6 h was associated with neovascular AMD (OR 3.09, 95% CI 1.20–7.97). A cross-sectional Mendelian randomization study exploring a potential causal relationship between risk factors and AMD also suggested that shorter sleep duration was correlated with an increased risk of AMD (OR 1.364, *p* = 0.036) [85]

A different cohort study, which followed 108,255 subjects (2094 diagnosed with AMD) for a mean of approximately 5 years, demonstrated that patients with insomnia were more likely to develop AMD compared to those without insomnia (2.5% versus 1.8%, *p* < 0.001, HR 1.33), and the incidence of exudative AMD was also higher in the population with insomnia (0.3% vs. 0.2%, *p* = 0.002, HR 1.67) [86]. A separate study followed 1003 consecutive patients and concluded that after controlling for age, gender, and smoking, sleep was not associated with neovascular AMD (*p* = 0.97), but interestingly, sleeping >8 h was associated with GA (the OR of 7.09) compared to patients without AMD [87]. Yet another study found suggestive evidence with marginal significance for the association of sleep duration with AMD and early AMD [88].

Thus, although some observational studies have found a strong correlation between increased risk of AMD and sleep deficiency, there are insufficient data to make this conclusion and no explanation for a proposed mechanism to explain this phenomenon. Some studies suggest theoretical mechanisms, such as decreased melatonin synthesis, which is involved in the control of the sleep–wake cycle and has been linked with retinal cell functionality; however, none have been experimentally proven [89].

Table 2 lists the important studies mentioned in this paper relating to risk factors associated with age-related macular degeneration. The following are abbreviations used in the table: HR = Hazards Ratio, OR = Odds Ratio, CI = Confidence interval, AMD = age-related macular degeneration, GA = geographic atrophy, DHA = docosahexaenoic acid, EPA = eicosapentaenoic acid, AREDS = Age-Related Eye Disease Studies, RR = relative risk, BMI = body mass index.

## 15. Conclusions

Identifying the interactions among diet, gut microbiota, vitamin supplementation, overall health, physical activity, genotype, BMI, and sleep patterns represents a new frontier in treating various metabolic diseases, including AMD. This understanding paves the way for proactive interventions and potentially personalized treatment strategies in the future.

AMD is a multifaceted condition involving intricate interactions between lifestyle, systemic health, and genetic variables. Oxidative stress and inflammation are among the main factors in the pathophysiology of AMD. The contribution of tobacco smoking, nutrition, exercise, and systemic illnesses, including diabetes, high blood pressure, obesity, and heart disease, highlights the importance of lifestyle choices for the modification of systemic and ocular health. These systemic factors may increase the risk of AMD by causing vascular damage, inflammation, and oxidative stress in the retina.

Current research suggests that the underlying pathologic processes can be modified by leading a healthy lifestyle that includes regular exercise, a balanced diet rich in antioxidants, taking AREDS2 supplements as appropriate, and avoiding smoking. In particular, the Mediterranean diet has shown promising results in lowering the risk of AMD progression, which may be further enhanced by specific, yet-to-be-discovered genetic predispositions. AREDS2 formulation is beneficial for reducing the risk of progression from dry to wet form of AMD and may be beneficial for reducing the risk of GA progression toward the central macula, as the newer analyses appear to show. Currently, genetic testing is not recommended, as data supporting its benefit in predicting disease progression or management of AMD are lacking.

## 16. Future Directions

AMD has a complex etiology that requires a multimodal approach involving the study of genetic predispositions, management of systemic health variables, and promotion of a healthy lifestyle. Further research is needed to decipher interactions between various risk factors with an overarching goal of developing more effective care and prevention strategies for individuals at risk of AMD. Continued collaboration among ophthalmologists, dietitians, geneticists, and basic scientists worldwide underscores a shared commitment to unraveling the complexities of AMD and providing hope for more sophisticated prevention paradigms and individualized treatment strategies for those at risk or affected by this condition.

## Figures and Tables

**Table 1:** The Age-Related Eye Disease Study 2 Supplement Daily Dose.

Vitamin C	500 mg
Vitamin E	400 international units
Lutein	10 mg
Zeaxanthin	2 mg
Zinc (oxide)	80 mg
Copper (cupric oxide)	2 mg

**Table 2:** Summary of Important Risk Factors Associated with AMD.

Study	Study Design	Sample Size	Country	Odds Ratio/Hazards Ratio (95% CI)	Key Findings
Merle et al. [9]	Longitudinal	2525	USA	HR 0.74 (95% CI 0.61–0.91); *p*-trend = 0.007	High adherence to Mediterranean diet reduced progression to advanced AMD.
Merle et al. [21]	Longitudinal	4996	The Netherland, France	HR 0.59 (95% CI 0.37–0.95); *p* = 0.04	Mediterranean diet adherence reduced late AMD risk by 41%.
Chiu et al. [24]	Cross-Sectional	4088	USA	Oriental: OR= 0.74 (95% CI 0.59–0.91; *p* = 0.01) for early AMD and 0.38 (0.27–0.54; *p* < 0.0001) for advanced AMD. Western: OR = 1.56 (1.18–2.06; *p* = 0.01) for early AMD and 3.70 (2.31–5.92; *p* < 0.0001) for advanced AMD	Western diet increased AMD risk; Mediterranean-like diet reduced early and advanced AMD risk.
Mares et al. [23]	Observational cohort	1325	USA	Women in the highest, compared to the lowest, quintile for modified healthy eating index score had 46% lowering of odds for early AMD multivariate-adjusted OR (95% CI) = 0.54 (0.33–0.88)	High scores on Mediterranean diet scale correlated with lower AMD rates (6-year follow-up).
AREDS Group [27]	Longitudinal	3609	USA	Statistically significant odds reduction in the development of advanced AMD with antioxidants plus zinc (odds ratio [OR], 0.72; 99% confidence interval [CI], 0.52–0.98). ORs for zinc alone and antioxidants alone were 0.75 (99% CI, 0.55–1.03) and 0.80 (99% CI, 0.59–1.09), respectively. Subjects with the complement factor H gene rs10922109 mutation who followed a Mediterranean diet were less likely to develop late AMD (*p* = 0.01), GA (*p* = 0.003), and neovascular AMD (*p* = 0.026) than those who followed a standard diet.	Individuals with extensive intermediate-sized drusen, at least one large drusen, noncentral geographic atrophy in one or both eyes, or advanced AMD or vision loss from AMD in one eye, and who do not have contraindications like smoking, should consider using antioxidant and zinc supplements.
Chew et al. [28]	Longitudinal	4203	USA	Lutein/zeaxanthin provided a significantly stronger protective effect against progression to late AMD (HR 0.82, *p* = 0.02), particularly for the development of neovascular AMD (HR 0.78, *p* = 0.01) but not for the development of central GA (HR 0.94, *p* = 0.67). More lung cancer cases in the beta-carotene group compared to the group without beta-carotene (2.0% vs. 0.9%, *p* = 0.04)	The AREDS2 study concluded that, although adding lutein plus zeaxanthin, DHA plus EPA, or both to the original AREDS formulation did not show additional benefits, lutein and zeaxanthin could replace beta-carotene in the original AREDS formulation to reduce the risk of lung cancer among smokers.
Alsoudi et al. [34]	Retrospective cohort	66,804	USA	A 77% risk reduction in developing nonexudative AMD (relative risk [RR] 0.23; *p* < 0.001), an 89% reduction in developing advanced nonexudative AMD with GA (RR 0.11; *p* < 0.001), a 72% reduction in developing exudative AMD (RR 0.28; *p* < 0.001 in those taking curcuma-based supplements	Consumption of curcuma-based nutritional supplements was found to be associated with a lower risk of developing age-related macular degeneration
Klein et al. [41]	Longitudinal	4926	USA	Current smokers had an increased risk of early AMD (OR: 1.47, *p* = 0.01) and AMD progression (OR: 1.43, *p* = 0.02) Association between time spent outside and the development of early AMD ((OR 2.26; 95% CI 1.06–4.81))	Current smoking was associated with a 45% increased odds of developing early AMD or AMD progression. Increased time spent outside could also be associated with an increased risk of early AMD.
McGuinnes et al. [45]	Meta-analysis	In results	US, Europe, Australia	Regular moderate physical activity was related to rates of both early AMD (8 studies, *n* = 38,112, OR 0.92, 95% CI 0.86–0.98) and late AMD (7 studies, *n* = 28,854, OR 0.59, 95% CI 0.49–0.72)	Moderate physical activity could reduce risks of early AMD and late AMD.
Hwang et al. [54]	Population-based cohort study	1,768,018 diabetics	South Korea	Diabetes of 5 or more years had a greater risk of developing AMD with an HR of 1.13 (95% CI 1.07–1.18). Furthermore, insulin use (HR 1.16) and the presence of vision-threatening diabetic retinopathy (HR = 1.40) were associated with an increased risk of exudative AMD, particularly in those less than 65 years of age.	A longer duration of diabetes, the use of insulin for diabetes management, and the presence of vision-threatening diabetic retinopathy were linked to a higher likelihood of developing exudative AMD.
Adams et al. [63]	Prospective cohort	21,287	Australia	Abdominal obesity was an independent risk factor in men for early AMD (OR 1.13; 95% CI 1.01–1.26; *p* = 0.03) and late AMD (OR 1.75; 95% CI 1.11–2.76; *p* = 0.02). In women, there was an inverse association for early AMD with all adipose measures studied (OR 0.89–0.93, *p* = 0.002–0.02) and no association for late AMD.	Obesity and higher body mass index (BMI) were associated with an elevated risk of early and late AMD in men.
Tsai et al. [82]	Population-based cohort	108,255	Taiwan	Insomnia patients were more likely to develop AMD compared to those without insomnia (2.5% versus 1.8%, *p* < 0.001, HR 1.33), and the incidence of exudative AMD was also higher in the population with insomnia (0.3% versus 0.2%, *p* = 0.002, HR 1.67)	Insomnia patients were more likely to develop AMD compared to those without insomnia.
Khurana et al. [83]	Observational	1003	USA	After controlling for age, gender, and smoking, sleep was not associated with neovascular AMD (*p* = 0.97), but interestingly, sleeping >8 h was associated with GA (the OR of 7.09) compared to patients without AMD	Sleep was not associated with an increased risk of developing neovascular AMD, but sleeping >8 h was associated with GA.

## Data Availability

No new data were created or analyzed in this study.

## References

[1] Deng Y., Qiao L., Du M., Qu C., Wan L., Li J., Huang L. (2022). Age-related macular degeneration: Epidemiology, genetics, pathophysiology, diagnosis, and targeted therapy. Genes. Dis..

[2] Vyawahare H., Shinde P. (2022). Age-Related Macular Degeneration: Epidemiology, Pathophysiology, Diagnosis, and Treatment. Cureus.

[3] Murkey S.P., Agarwal A., Pandit P., Kumar S., Jaiswal A. (2023). Unveiling the Spectrum of Ophthalmic Manifestations in Nutritional Deficiencies: A Comprehensive Review. Cureus.

[4] Juan C.A., Pérez de la Lastra J.M., Plou F.J., Pérez-Lebeña E. (2021). The Chemistry of Reactive Oxygen Species (ROS) Revisited: Outlining Their Role in Biological Macromolecules (DNA, Lipids and Proteins) and Induced Pathologies. Int. J. Mol. Sci..

[5] Kurihara T., Lee D., Shinojima A., Kinoshita T., Nishizaki S., Arita Y., Hidaka Y., Nishi Y., Shirakawa Y., Kimura S. (2021). Glucose levels between the anterior chamber of the eye and blood are correlated based on blood glucose dynamics. PLoS ONE.

[6] Wangsa-Wirawan N.D., Linsenmeier R.A. (2003). Retinal oxygen: Fundamental and clinical aspects. Arch. Ophthalmol..

[7] Martínez Leo E.E., Peñafiel A.M., Hernández Escalante V.M., Cabrera Araujo Z.M. (2021). Ultra-processed diet, systemic oxidative stress, and breach of immunologic tolerance. Nutrition.

[8] Wang J., Li M., Geng Z., Khattak S., Ji X., Wu D., Dang Y. (2022). Role of Oxidative Stress in Retinal Disease and the Early Intervention Strategies: A Review. Oxid. Med. Cell Longev..

[9] Merle B.M.J., Silver R.E., Rosner B., Seddon J.M. (2015). Adherence to a Mediterranean diet, genetic susceptibility, and progression to advanced macular degeneration: A prospective cohort study. Am. J. Clin. Nutr..

[10] Chiu C.J., Milton R.C., Klein R., Gensler G., Taylor A. (2007). Dietary carbohydrate and the progression of age-related macular degeneration: A prospective study from the Age-Related Eye Disease Study. Am. J. Clin. Nutr..

[11] Heesterbeek T.J., Lorés-Motta L., Hoyng C.B., Lechanteur Y.T.E., den Hollander A.I. (2020). Risk factors for progression of age-related macular degeneration. Ophthalmic Physiol. Opt..

[12] Witek K., Wydra K., Filip M. (2022). A High-Sugar Diet Consumption, Metabolism and Health Impacts with a Focus on the Development of Substance Use Disorder: A Narrative Review. Nutrients.

[13] Holesh J.E., Aslam S., Martin A. Physiology, Carbohydrates. StatPearls [Internet], Updated on 12 May 2023. https://www.ncbi.nlm.nih.gov/books/NBK459280/.

[14] Fleckenstein M., Keenan T.D.L., Guymer R.H., Chakravarthy U., Schmitz-Valckenberg S., Klaver C.C., Wong W.T., Chew E.Y. (2021). Age-related macular degeneration. Nat. Rev. Dis. Primers.

[15] Kaarniranta K., Uusitalo H., Blasiak J., Felszeghy S., Kannan R., Kauppinen A., Salminen A., Sinha D., Ferrington D. (2020). Mechanisms of mitochondrial dysfunction and their impact on age-related macular degeneration. Prog. Retin. Eye Res..

[16] Gaesser G.A. (2020). Whole Grains, Refined Grains, and Cancer Risk: A Systematic Review of Meta-Analyses of Observational Studies. Nutrients.

[17] WebMD (2023). What’s the Difference Between Good and Bad Carbs?. https://www.webmd.com/diet/whats-the-difference-between-good-and-bad-carbs.

[18] Harvard T.H. (2023). Chan School of Public Health. Carbohydrates and Blood Sugar. The Nutrition Source. https://nutritionsource.hsph.harvard.edu/carbohydrates/carbohydrates-and-blood-sugar/.

[19] Harvard Health Publishing (2019). Are Certain Types of Sugars Healthier Than Others? Harvard Health Blog. https://www.health.harvard.edu/blog/are-certain-types-of-sugars-healthier-than-others-2019052916699.

[20] Guasch-Ferré M., Willett W.C. (2021). The Mediterranean diet and health: A comprehensive overview. J. Intern. Med..

[21] Merle B.M.J., Colijn J.M., Cougnard-Grégoire A., de Koning-Backus A.P.M., Delyfer M.-N., Kiefte-de Jong J.C., Meester-Smoor M., Féart C., Verzijden T., Samieri C. (2019). Mediterranean Diet and Incidence of Advanced Age-Related Macular Degeneration: The EYE-RISK Consortium. Ophthalmology.

[22] Willett W.C., Sacks F., Trichopoulou A., Drescher G., Ferro-Luzzi A., Helsing E., Trichopoulos D. (1995). Mediterranean diet pyramid: A cultural model for healthy eating. Am. J. Clin. Nutr..

[23] Mares J.A., Voland R.P., Sondel S.A., Millen A.E., Larowe T., Moeller S.M., Klein M.L., Blodi B.A., Chappell R.J., Tinker L. (2011). Healthy lifestyles related to subsequent prevalence of age-related macular degeneration. Arch. Ophthalmol..

[24] Chiu C.-J., Chang M.-L., Zhang F.F., Li T., Gensler G., Schleicher M., Taylor A. (2014). The relationship of major American dietary patterns to age-related macular degeneration. Am. J. Ophthalmol..

[25] Keenan T.D., Agrón E., Mares J., Clemons T.E., van Asten F., Swaroop A., Chew E.Y. (2020). Adherence to the Mediterranean Diet and Progression to Late Age-Related Macular Degeneration in the Age-Related Eye Disease Studies 1 and 2. Ophthalmology.

[26] Chew E.Y., Clemons T.E., Agrón E., Domalpally A., Keenan T.D.L., Vitale S., Weber C., Smith D.C., Christen W. (2022). Long-term Outcomes of Adding Lutein/Zeaxanthin and ω-3 Fatty Acids to the AREDS Supplements on Age-Related Macular Degeneration Progression: AREDS2 Report 28. JAMA Ophthalmol..

[27] Age-Related Eye Disease Study Research Group (2001). A randomized, placebo-controlled, clinical trial of high-dose supplementation with vitamins C and E, beta carotene, and zinc for age-related macular degeneration and vision loss: AREDS report no. 8. Arch. Ophthalmol..

[28] Chew E.Y., Clemons T., SanGiovanni J.P., Danis R., Domalpally A., McBee W., Sperduto R., Ferris F.L. (2012). The Age-Related Eye Disease Study 2 (AREDS2): Study design and baseline characteristics (AREDS2 report number 1). Ophthalmology.

[29] Age-Related Eye Disease Study 2 (AREDS2) Research Group (2013). Lutein + zeaxanthin and omega-3 fatty acids for age-related macular degeneration: The Age-Related Eye Disease Study 2 (AREDS2) randomized clinical trial. JAMA.

[30] Chew E.Y., Clemons T.E., Sangiovanni J.P., Danis R.P., Ferris F.L., Elman M.J., Antoszyk A.N., Ruby A.J., Orth D., Bressler S.B. (2014). Secondary analyses of the effects of lutein/zeaxanthin on age-related macular degeneration progression: AREDS2 report No. 3. JAMA Ophthalmol..

[31] Lindblad A.S., Lloyd P.C., Clemons T.E., Gensler G.R., Ferris F.L., Klein M.L., Armstrong J.R. (2009). Change in area of geographic atrophy in the Age-Related Eye Disease Study: AREDS report number 26. Arch. Ophthalmol..

[32] Keenan T.D.L., Agrón E., Keane P.A., Domalpally A., Chew E.Y. (2025). Oral Antioxidant and Lutein/Zeaxanthin Supplements Slow Geographic Atrophy Progression to the Fovea in Age-Related Macular Degeneration. Ophthalmology.

[33] Heier J.S., Lad E.M., Holz F.G., Rosenfeld P.J., Guymer R.H., Boyer D., Grossi F., Baumal C.R., Korobelnik J.-F., Slakter J.S. (2023). Pegcetacoplan for the treatment of geographic atrophy secondary to age-related macular degeneration (OAKS and DERBY): Two multicentre, randomised, double-masked, sham-controlled, phase 3 trials. Lancet.

[34] Eigner D., Scholz D. (1999). Ferula asa-foetida and Curcuma longa in traditional medical treatment and diet in Nepal. J. Ethnopharmacol..

[35] Woo J.M., Shin D.-Y., Lee S.J., Joe Y., Zheng M., Yim J.H., Callaway Z., Chung H.T. (2012). Curcumin protects retinal pigment epithelial cells against oxidative stress via induction of heme oxygenase-1 expression and reduction of reactive oxygen. Mol. Vis..

[36] Alsoudi A.F., Wai K.M., Koo E., Mruthyunjaya P., Rahimy E. (2024). Curcuma-Based Nutritional Supplements and Risk of Age-Related Macular Degeneration. JAMA Ophthalmol..

[37] Allegrini D., Raimondi R., Angi M., Ricciardelli G., Montericcio A., Borgia A., Romano M.R. (2021). Curcuma-Based Nutritional Supplement in Patients with Neovascular Age-Related Macular Degeneration. J. Med. Food.

[38] Chew E.Y. (2020). Age-related Macular Degeneration: Nutrition, Genes and Deep Learning-The LXXVI Edward Jackson Memorial Lecture. Am. J. Ophthalmol..

[39] Suñer I.J., Espinosa-Heidmann D.G., Marin-Castano M.E., Hernandez E.P., Pereira-Simon S., Cousins S.W. (2004). Nicotine increases size and severity of experimental choroidal neovascularization. Investig. Ophthalmol. Vis. Sci..

[40] Blasiak J., Petrovski G., Veréb Z., Facskó A., Kaarniranta K. (2014). Oxidative stress, hypoxia, and autophagy in the neovascular processes of age-related macular degeneration. Biomed. Res. Int..

[41] Seddon J.M., Willett W.C., Speizer F.E., Hankinson S.E. (1996). A prospective study of cigarette smoking and age-related macular degeneration in women. JAMA.

[42] Christen W.G., Glynn R.J., Manson J.E., Ajani U.A., Buring J.E. (1996). A prospective study of cigarette smoking and risk of age-related macular degeneration in men. JAMA.

[43] Klein R., Knudtson M.D., Cruickshanks K.J., Klein B.E.K. (2008). Further observations on the association between smoking and the long-term incidence and progression of age-related macular degeneration: The Beaver Dam Eye Study. Arch. Ophthalmol..

[44] Tomany S.C., Wang J.J., Van Leeuwen R., Klein R., Mitchell P., Vingerling J.R., Klein B.E.K., Smith W., De Jong P.T.V.M. (2004). Risk factors for incident age-related macular degeneration: Pooled findings from 3 continents. Ophthalmology.

[45] Klein R., Cruickshanks K.J., Nash S.D., Krantz E.M., Nieto F.J., Huang G.H., Pankow J.S., Klein B.E.K. (2010). The prevalence of age-related macular degeneration and associated risk factors. Arch. Ophthalmol..

[46] Radak Z., Taylor A.W., Ohno H., Goto S. (2001). Adaptation to exercise-induced oxidative stress: From muscle to brain. Exerc. Immunol. Rev..

[47] McGuinness M.B., Le J., Mitchell P., Gopinath B., Cerin E., Saksens N.T.M., Schick T., Hoyng C.B., Guymer R.H., Finger R.P. (2017). Physical Activity and Age-related Macular Degeneration: A Systematic Literature Review and Meta-analysis. Am. J. Ophthalmol..

[48] Makin R.D., Argyle D., Hirahara S., Nagasaka Y., Zhang M., Yan Z., Kerur N., Ambati J., Gelfand B.D. (2020). Voluntary Exercise Suppresses Choroidal Neovascularization in Mice. Invest. Ophthalmol. Vis. Sci..

[49] Chalam K.V., Khetpal V., Rusovici R., Balaiya S. (2011). A review: Role of ultraviolet radiation in age-related macular degeneration. Eye Contact Lens.

[50] Rózanowska M., Korytowski W., Rózanowski B., Skumatz C., Boulton M.E., Burke J.M., Sarna T. (2002). Photoreactivity of aged human RPE melanosomes: A comparison with lipofuscin. Investig. Ophthalmol. Vis. Sci..

[51] CTomany S.C., Cruickshanks K.J., Klein R., Klein B.E., Knudtson M.D. (2001). Sunlight and the 5-year incidence of early age-related maculopathy: The beaver dam eye study. Arch. Ophthalmol..

[52] Hyman L., Schachat A.P., He Q., Leske M.C. (2000). Hypertension, cardiovascular disease, and age-related macular degeneration. Age-Related Macular Degeneration Risk Factors Study Group. Arch. Ophthalmol..

[53] Lee J., Suh H.S., Hwang I.C. (2021). The Relationship between Age-Related Macular Degeneration and Cardiovascular Disease: A Meta-Analysis. Iran J. Public Health.

[54] Klein R., Deng Y., Klein B.E.K., Hyman L., Seddon J., Frank R.N., Wallace R.B., Hendrix S.L., Kuppermann B.D., Langer R.D. (2007). Cardiovascular disease, its risk factors and treatment, and age-related macular degeneration: Women’s Health Initiative Sight Exam ancillary study. Am. J. Ophthalmol..

[55] Chang C.-C., Huang C.-H., Chou Y.-C., Chang J.-Y., Sun C.-A. (2021). Association Between Age-Related Macular Degeneration and Risk of Heart Failure: A Population-Based Nested Case-Control Study. J. Am. Heart Assoc..

[56] Hwang S., Kang S.W., Kim S.J., Lee K.N., Han K., Lim D.H. (2023). Diabetes-Related Risk Factors for Exudative Age-Related Macular Degeneration: A Nationwide Cohort Study of a Diabetic Population. Investig. Ophthalmol. Vis. Sci..

[57] Leske M.C., Wu S.-Y., Hennis A., Nemesure B., Yang L., Hyman L., Schachat A.P. (2006). Nine-year incidence of age-related macular degeneration in the Barbados Eye Studies. Ophthalmology.

[58] Clemons T.E., Milton R.C., Klein R., Seddon J.M., Ferris F.L. (2005). 3rd. Risk factors for the incidence of Advanced Age-Related Macular Degeneration in the Age-Related Eye Disease Study (AREDS) AREDS report no. 19. Ophthalmology.

[59] Klein R., Klein B.E., Moss S.E. (1992). Diabetes, hyperglycemia, and age-related maculopathy. Ophthalmology.

[60] Topouzis F., Anastasopoulos E., Augood C., Bentham G.C., Chakravarthy U., de Jong P.T.V.M., Rahu M., Seland J., Soubrane G., Tomazzoli L. (2009). Association of diabetes with age-related macular degeneration in the EUREYE study. Br. J. Ophthalmol..

[61] Johnson E.J. (2005). Obesity, lutein metabolism, and age-related macular degeneration: A web of connections. Nutr. Rev..

[62] Klein R., Klein B.E.K., Tomany S.C., Cruickshanks K.J. (2003). The association of cardiovascular disease with the long-term incidence of age-related maculopathy: The Beaver Dam Eye Study. Ophthalmology.

[63] Zhang Q.-Y., Tie L.-J., Wu S.-S., Lv P.-L., Huang H.-W., Wang W.-Q., Wang H., Ma L. (2016). Overweight, Obesity, and Risk of Age-Related Macular Degeneration. Investig. Ophthalmol. Vis. Sci..

[64] Peeters A., Magliano D.J., Stevens J., Duncan B.B., Klein R., Wong T.Y. (2008). Changes in abdominal obesity and age-related macular degeneration: The Atherosclerosis Risk in Communities Study. Arch. Ophthalmol..

[65] Adams M.K.M., Simpson J.A., Aung K.Z., Makeyeva G.A., Giles G.G., English D.R., Hopper J., Guymer R.H., Baird P.N., Robman L.D. (2011). Abdominal obesity and age-related macular degeneration. Am. J. Epidemiol..

[66] Blum H.E. (2017). The human microbiome. Adv. Med. Sci..

[67] Devkota S., Wang Y., Musch M.W., Leone V., Fehlner-Peach H., Nadimpalli A., Antonopoulos D.A., Jabri B., Chang E.B. (2012). Dietary-fat-induced taurocholic acid promotes pathobiont expansion and colitis in Il10-/- mice. Nature.

[68] Wang B., Yao M., Lv L., Ling Z., Li L. (2017). The Human Microbiota in Health and Disease. Engineering.

[69] Lin P., McClintic S.M., Nadeem U., Skondra D. (2021). A Review of the Role of the Intestinal Microbiota in Age-Related Macular Degeneration. J. Clin. Med..

[70] Zinkernagel M.S., Zysset-Burri D.C., Keller I., Berger L.E., Leichtle A.B., Largiadèr C.R., Fiedler G.M., Wolf S. (2017). Association of the Intestinal Microbiome with the Development of Neovascular Age-Related Macular Degeneration. Sci. Rep..

[71] Nadeem U., Xie B., Movahedan A., D’Souza M., Barba H., Deng N., Leone V.A., Chang E., Sulakhe D., Skondra D. High throughput RNA sequencing of germ-free mouse retina reveals metabolic pathways involved in the gut-retina axis. bioRxiv.

[72] Zhang J.Y., Xie B., Barba H., Nadeem U., Movahedan A., Deng N., Spedale M., D’Souza M., Luo W., Leone V. (2022). Absence of Gut Microbiota Is Associated with RPE/Choroid Transcriptomic Changes Related to Age-Related Macular Degeneration Pathobiology and Decreased Choroidal Neovascularization. Int. J. Mol. Sci..

[73] Clarke S.F., Murphy E.F., Nilaweera K., Ross P.R., Shanahan F., O’Toole P.W., Cotter P.D. (2012). The gut microbiota and its relationship to diet and obesity: New insights. Gut Microbes.

[74] Dao D., Xie B., Nadeem U., Xiao J., Movahedan A., D’Souza M., Leone V., Hariprasad S.M., Chang E.B., Sulakhe D. (2021). High-Fat Diet Alters the Retinal Transcriptome in the Absence of Gut Microbiota. Cells.

[75] Xiao J., Xie B., Dao D., Spedale M., D’Souza M., Theriault B., Hariprasad S.M., Sulakhe D., Chang E.B., Skondra D. (2022). High-Fat Diet Alters the Retinal Pigment Epithelium and Choroidal Transcriptome in the Absence of Gut Microbiota. Cells.

[76] Rowan S., Jiang S., Korem T., Szymanski J., Chang M.-L., Szelog J., Cassalman C., Dasuri K., McGuire C., Nagai R. (2017). Involvement of a gut-retina axis in protection against dietary glycemia-induced age-related macular degeneration. Proc. Natl. Acad. Sci. USA.

[77] Singh M., Tyagi S.C. (2018). *;* Tyagi, S.C. Genes and genetics in eye diseases: A genomic medicine approach for investigating hereditary and inflammatory ocular disorders. Int. J. Ophthalmol..

[78] Shahid H., Khan J.C., Cipriani V., Sepp T., Matharu B.K., Bunce C., Harding S.P., Clayton D.G., Moore A.T., Yates J.R.W. (2012). Age-related macular degeneration: The importance of family history as a risk factor. Br. J. Ophthalmol..

[79] Bhumika, Bora N.S., Bora P.S. (2024). Genetic Insights into Age-Related Macular Degeneration. Biomedicines.

[80] Cebatoriene D., Vilkeviciute A., Gedvilaite-Vaicechauskiene G., Duseikaite M., Bruzaite A., Kriauciuniene L., Zaliuniene D., Liutkeviciene R. (2024). The Impact of ARMS2 (rs10490924), VEGFA (rs3024997), TNFRSF1B (rs1061622), TNFRSF1A (rs4149576), and IL1B1 (rs1143623) Polymorphisms and Serum Levels on Age-Related Macular Degeneration Development and Therapeutic Responses. Int. J. Mol. Sci..

[81] Ayala-Haedo J.A., Gallins P.J., Whitehead P.L., Schwartz S.G., Kovach J.L., Postel E.A., Agarwal A., Wang G., Haines J.L., Pericak-Vance M.A. (2010). Analysis of single nucleotide polymorphisms in the NOS2A gene and interaction with smoking in age-related macular degeneration. Ann. Hum. Genet..

[82] Vilkeviciute A., Pileckaite E., Bruzaite A., Cebatoriene D., Gedvilaite-Vaicechauskiene G., Kriauciuniene L., Zaliuniene D., Liutkeviciene R. (2024). Evaluating *TAB2, IKBKB,* and *IKBKG* Gene Polymorphisms and Serum Protein Levels and Their Association with Age-Related Macular Degeneration and Its Treatment Efficiency. Medicina.

[83] Devi S.M., Mahalaxmi I., Kaavya J., Chinnkulandhai V., Balachandar V. (2021). Does epigenetics have a role in age related macular degeneration and diabetic retinopathy?. Genes Dis..

[84] Pérez-Canales J.L., Rico-Sergado L., Pérez-Santonja J.J. (2016). Self-Reported Sleep Duration in Patients with Neovascular Age-Related Macular Degeneration. Ophthalmic. Epidemiol..

[85] Lei S., Liu Z., Li H. (2023). Sleep duration and age-related macular degeneration: A cross-sectional and Mendelian randomization study. Front. Aging Neurosci..

[86] Tsai D.-C., Chen H.-C., Leu H.-B., Chen S.-J., Hsu N.-W., Huang C.-C., Chen J.-W., Lin S.-J., Chou P. (2020). The association between clinically diagnosed insomnia and age-related macular degeneration: A population-based cohort study. Acta Ophthalmol..

[87] Khurana R.N., Porco T.C., Claman D.M., Boldrey E.E., Palmer J.D., Wieland M.R. (2016). Increasing sleep duration is associated with geographic atrophy and age-related macular degeneration. Retina.

[88] Zhu R.-C., Li F.-F., Wu Y.-Q., Yi Q.-Y., Huang X.-F. (2023). Minimal effect of sleep on the risk of age-related macular degeneration: A Mendelian randomization study. Front. Aging Neurosci..

[89] Ostrin L.A. (2019). Ocular and systemic melatonin and the influence of light exposure. Clin. Exp. Optom..

